# Astrocyte heterogeneity and hierarchical information flow: reframing glial computation

**DOI:** 10.3389/fncel.2026.1843853

**Published:** 2026-06-17

**Authors:** Oswaldo Pérez, James Schummers, Mónica López-Hidalgo

**Affiliations:** 1 Escuela Nacional de Estudios Superiores, Unidad Juriquilla, Universidad Nacional Autónoma de México, Querétaro, México; 2 Department of Biomedical Engineering, Florida International University, Miami, FL, United States

**Keywords:** Astrocyte heterogeneity, hierarchical information flow, glial computation, calcium signaling, cellular polarity, graph theory analysis

## Abstract

Astrocytes are active elements in the brain; they exhibit complex, highly branched processes that contact neurons and blood vessels to regulate neuronal activity and vasoactive responses. Distal processes receive information that is integrated in the soma; however, rather than uniform units, astrocytes display structural and functional heterogeneity across their processes. Furthermore, it remains unclear whether all the distal processes serve as input sites, output sites, or whether specific domains contribute more strongly to somatic responses. In this Perspective article, we discuss this hierarchical organization of information flow, with specialized compartments acting as input and output elements while others play integrating roles. Here, we discuss that astrocyte polarity depends on the local circuit and is dynamically reshaped by development, aging, and disease. Graph-theoretical approaches support hierarchical network structures within astrocytes for processing information and open the door to their contribution to brain computation in physiological and pathological reconfiguration.

## Introduction

Cellular polarity refers to the asymmetric distribution of cellular components that create different functional domains within the cell. This asymmetry is crucial to accomplish multiple cellular functions and to have directional information flow. This is exemplified in polarized epithelial cells such as intestinal enterocytes, where the apical surface, with a brush border of microvilli, faces the intestinal lumen for nutrient absorption, while the basolateral surface interacts with the bloodstream for nutrient transport (Piroli et al., 2019). Neurons are polarized cells, with signal-receiving dendrites that determine the amount of integrated information, and signal-transmitting axons that target neurons and determine the direction of information flow (Dieters, 1863; Banker, 2018). For instance, visual cortical pyramidal neurons extend their apical dendrites vertically to receive columnar inputs, and their basal dendrites laterally integrate distinct features from neighboring columns for visual processing. In contrast, their single axon projects to numerous cortical and subcortical regions (Hübener et al., 1990). Similarly, primary afferents neurons extend their processed to the periphery, acing as dendrites, and their axon contacts neurons of the dorsal horn of the spinal cord, ensuring unidirectional sensory flow (Tandrup, 1995). Even in local interneurons, polarized synapse distribution on dendrites and axons enables precise circuit control (Zhang et al., 2024).

In contrast to the well-established polarity of neurons, astrocytes polarity is less understood. Astrocytes exhibit complex, highly branched processes that contact neurons with few specialize processes reaching blood vessels (end foot), the ventricles or the pial surfaces (Derouiche et al., 2012). Their branching complexity and the diversity of astrocytic shapes are context-dependent, varying across brain regions and even species. For example, carnivores display more elaborate astrocyte morphologies than rodents (López-Hidalgo et al., 2016). Ferret visual cortical astrocytes are approximately twice the size of their mouse counterparts and exhibit more complex shapes, including increased invaginations and elongated processes, compared to the more spherical and homogenous mouse astrocytes (López-Hidalgo et al., 2016). Human interlaminar astrocytes exhibit a modular organization aligned to the columnar structure of the cortex. Their cell bodies are located in layer I and their long processes extend into deeper cortical layers (Falcone et al., 2019) enabling integration of information across cortical layers. A central question in understanding astrocyte polarity is whether there exists a directional flow of information within the astrocyte, with specific astrocyte compartments such as soma and processes, functioning as inputs, outputs, or central processing hubs.

## Are all the processes the same?

Astrocytic polarity depends on the distribution of molecular features that provide distinct capabilities to the processes, enabling it to act as input and output compartments. For instance, perivascular end feet are specialized processes involved in water and potassium homeostasis and are characterized by high expression of aquaporin-4 and Kir4.1 channels (Derouiche et al., 2012). In contrast, perisynaptic astrocytic processes display a selective repertoire of ionotropic, metabotropic receptors, and transporters to rapidly detect neuroactive substances and modulate synaptic transmission and plasticity (Bazargani and Attwell, 2016).

Beyond the specialization of membrane proteins, astrocyte polarity also depends on the intracellular distribution of organelles, which confers distinct functional identities to the distal processes or microdomains. For instance, the density of the endoplasmic reticulum varies across the processes, with a higher presence in distal perisynaptic. Similarly, mitochondria are non-randomly located and are dense where they have to meet metabolic demands. In perivascular endfeet, they form elaborate networks to support ion transport and water homeostasis, whereas in perisynaptic domains, mitochondrias buffer calcium transients and provide ATP during intense neuronal activity (Agarwal et al., 2017; Serrat et al., 2021).

The compartmental distribution of organelles not only sustains local metabolic and homeostatic demands but also gives rise to complex and diverse calcium dynamics across their compartments, allowing signaling to remain local or to extend to the whole cell and even across astrocytic networks (Losi et al., 2017; Cahill et al., 2024). In distal perisynaptic processes, calcium activity is typically fast and frequent, occurring within small, spatially restricted areas (Lia et al., 2021; Liu et al., 2023). This activity depends on the activity of ionotropic and metabotropic receptors as well as IP₃-mediated release from the endoplasmic reticulum and mitochondrial buffering in these fine structures (Serrat et al., 2021). These spontaneous signals are temporally precise and allow astrocytes to modulate local synaptic transmission (Semyanov et al., 2020).

In contrast, somatic calcium signals are slower, less frequent, and have larger amplitudes, reflecting their integrative role (Lia et al., 2021; Rusakov et al., 2025). These signals depend mainly on calcium release from internal stores and are regulated by local endoplasmic reticulum and mitochondria (Semyanov et al., 2020; Rusakov et al., 2025). Recently, it was shown that a spatial threshold of 23% of simultaneously active microdomains must be met for the soma of cortical astrocytes to activate (Lines et al., 2024). This highlights a functional division where distal processes process local synaptic activity, while the soma is a global integrator that coordinates whole-cell signals and synchronizes activity across astrocytic networks (Losi et al., 2017; Lia et al., 2021; Cahill et al., 2024).

While the data suggest a clear distinction between local activity in the processes and global activity induced by the soma, this is an oversimplification of how astrocytes process information and the flow of it within an astrocyte. For instance, it is still unclear whether all distal processes act as input sites or if there are specific domains that contribute more strongly to the somatic responses. Similarly, once somatic calcium activity is triggered, are all processes equally connected to the soma (output)? or do specific processes establish a directional flow of information toward neurons or vasculature? Does this organization depend on the underlying neural circuit, or is it intrinsic of astrocytes that is established during development? This raises the possibility of a hierarchical organization within astrocyte compartments, where certain processes may function as dominant inputs or outputs, while others would play more modulatory roles.

## Hierarchical organization and heterogeneity of astrocyte processes in information flow

**Figure 1:**
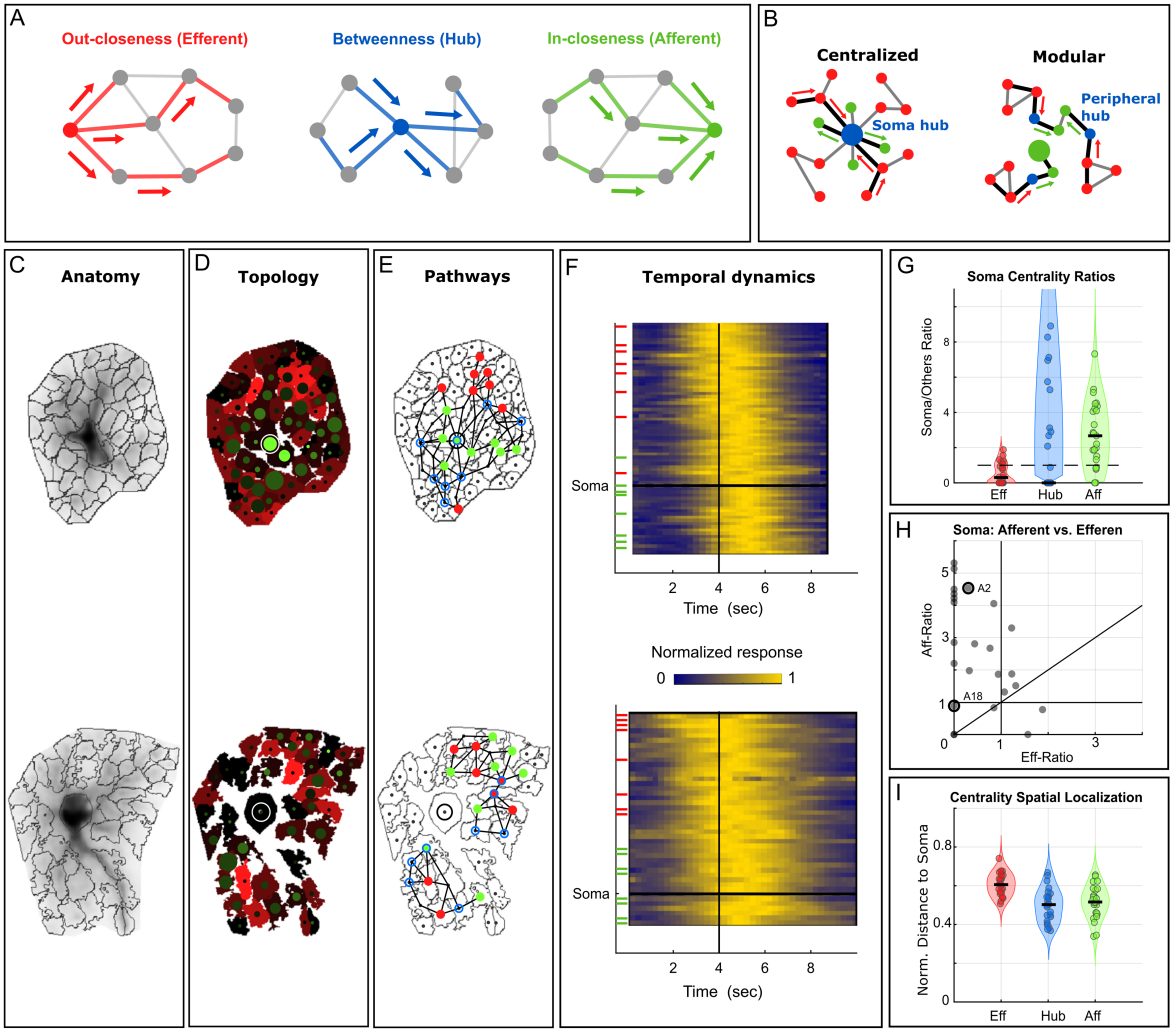
Functional architecture and information flow in astrocytes. **(A)** Schematic illustration of the three centrality metrics used for astrocyte processes. Out-closeness (efferent) delineates processes that initiate signals with short latencies, serving as efficient emitters. Betweenness (hub), identifies intermediary processes that bridge communication pathways. In-closeness (afferent) designates processes where signals conclude with prolonged latencies. **(B)** Primary architecture and dynamics of astrocytic communication. The centralized model, wherein the soma serves as the central hub, and the modular model, featuring clusters of peripheral processes as independent local hubs. In both architectures, the hierarchy involves a signal integration from efferent nodes toward hubs (red arrows), followed by the distribution of information toward afferent domains (green arrows). **(C)** Images of visual cortical astrocytes displaying regions of interest (ROIs) obtained using the algorithm described by Mukamel et al. (2009). **(D)** In-closeness (afferent), process network. Red shading denotes high out-closeness (efferent), green for high in-closeness (afferent), and node size indicates betweenness centrality (hubs). The soma is denoted by a black circle. **(E)** Optimal communication pathways. Red nodes represent efferent structures, green are afferent and blue are hubs. The black circle indicates the soma. **(F)** Temporal dynamics of calcium activity induced by visual stimulation (4 s, starting on 0 s. Stimulus offset is marked by vertical black line). Processes with high out-closeness (red marks) have responses with short latencies, whereas those exhibiting high in-closeness (green marks) were activated subsequently, displaying longer latencies. The soma is indicated by horizontal black line. **(G)** Soma centrality ratios. Distribution of the efferent-ratio (Eff), hub-ratio (Hub) and afferent-ratio (Aff). Each ratio is calculated by dividing the centrality value of soma by the average of the remaining processes. The hub ratio classifies astrocytic architectures as centralized (high value) or modular (low value, with local hubs) **(H)** Somatic afferent-efferent asymmetry. Scatter plot of the aff-ratio versus eff-ratio for individual somas. Most astrocytes display a somatic bias favoring signal afferent over efferent. **(I)** Centrality spatial localization. For each astrocyte, the weighted average of normalized distances from the soma was calculated. Distances were normalized between 0 (soma) and 1 (maximum process length), using the centrality measure of each process (Efferent, Eff; hub, Afferent, Aff) as the weight. Efferent processes predominate at distal locations, in contrast with hub and afferent processes, which are located at intermediates sites. Experimental data: calcium imaging data were obtained from visual cortical astrocytes of anesthetized ferrets. Data represents the transient peak responses recorded via two-photon microscopy during the presentation of 4 s drifting grating stimuli.

To investigate the functional architecture of astrocytes, graph theory metrics were applied to *in vivo* cortical astrocytes expressing GCaMP6s from anesthetized ferrets. We characterized the flow of information induced by 4 s visual stimulation (Figure 1) within individual astrocytes. Three metrics of the activity in the processes were assessed: out-closeness, which detects processes that act as signal initiators (efferent), with information outward to the rest of the astrocyte with short latencies; in-closeness, which identifies processes where information converges with long latencies (afferent); and betweenness, which detects intermediary processes linking communication routes at intermediate latencies (hub) (Figure 1A). The relative contribution of the soma versus the processes was quantified using centrality ratios. These were obtained by dividing the soma’s metric value by the average of that metric across the remaining processes within the astrocyte (eff-ratio, hub-ratio, aff-ratio). These ratios quantify the relative efficiency of the soma in signal emission, reception and intermediation compared to the processes (Figure 1G).

The hub ratio delineates two distinct organization models in astrocytes (Figures 1B,C). The first corresponds to a centralized model, wherein the soma serves as a hub that links distal efferent processes with central afferent processes. This configuration aligns with evidence that somatic activation requires the engagement of approximately 23% of astrocytic microdomains. On the other hand, a subset of astrocytes exhibits a modular model, defined by localized information fluxes that extend from efferent periphery to intermediate hubs and afferents (Figures 1D,E). This heterogeneity of astrocyte architecture underscores the specialized functional contributions of efferent, hub and afferent processes to local information processing demands.

This heterogeneity is evident in the temporal dynamics of astrocyte calcium responses. Our heatmaps reveal a temporal dynamic, in which the efferent processes that initiate the signals have a short delay, while afferent receiving them have a significantly longer lag (Figure 1F). Notably, the soma rarely acts as an efferent, instead, it functions as a flexible process, that fluctuates between hub and afferent signals (Figures 1G,H). This diversity in architecture suggests that each astrocyte process plays a distinct, specialized role in how the astrocyte integrates synaptic input before reaching its global activation.

Efferent, hub and afferent processes display a strategic spatial organization, with efferent processes situated slightly more distally from the soma (with an average normalized distance of ~0.6) relative to hub or afferent processes (average normalized distance ~0.5). In this context, the distance is normalized to the maximum radius of each astrocyte, where 0 corresponds to the soma and 1 to the most distal process. This heterogeneity highlights the functional dynamics within the astrocyte, whereby distal processes serve as sensors detecting local synaptic inputs. Accordingly, the astrocyte acts as a filter, synchronizing its own global calcium activity when the activity of peripheral processes exceeds an internal threshold.

This spatial configuration of information flow primarily reflects the initial centripetal integration of signals from distal efferent processes toward central hubs. However, the presence of afferent processes is also detected, suggesting a subsequent redistribution phase. Specifically, once information is integrated in the hubs, it can propagate back toward effector processes. Although the temporal window of our transient response analysis does not allow us to observe the full signaling cycle, the signal redistribution emanating from the hubs suggests a backpropagation mechanism or biphasic signaling. Accordingly, the soma or hubs operate not only as attractors but also as distributors of integrated information toward specific domains.

## Pathological reconfiguration of astrocytic information flow

The hierarchical organization can dynamically change in pathological conditions such as epilepsy or hyperexcitability. In these conditions, astrocytes undergo structural and functional rearrangements, such as mitochondrial fission, that produce smaller, and mobile mitochondria that are redistributed into the distal processes (Ko et al., 2016; Rodriguez Salazar et al., 2025). This allows astrocytes to handle calcium transients closer to synapses (in microdomains), potentially increasing the weight of afferent process and the spread of calcium signals. Also, there is an increase in oxidative stress and ROS production that increases the probability of InsP3R opening and the permeability of mitochondrial transition pores (Duchen, 2000; Agarwal et al., 2017) which can contribute to the amplification and propagation of calcium signals. Under this conditions, the distal processes can potentially reshape the hierarchy of astrocytic processes by changing the number and weight of afferent processes facilitating somatic activation shifting from a centralized configuration to a modular one. This could promote the soma to oversaturate and amplify irrelevant signals instead of suppressing them, reducing the response to important information.

Similarly, in aged mice, astrocytes undergo heterogeneous changes in calcium activity within their domains (Lalo et al., 2011, 2018; Verkhratsky, 2019; Ding et al., 2022). In the end feet, they display a reduction in calcium transients (Ding et al., 2022), and the expression of calreticulin (a calcium-handling protein), that deteriorates the blood–brain barrier integrity and neurovascular coupling (Zarate et al., 2023; Labarta-Bajo and Allen, 2025). In parallel, there is an age-dependent decline in the expression of glutamate transporters and a deficient glutamate clearance and K+ buffering (Verkhratsky and Semyanov, 2023). This means that while end feet lose calcium activity, primary branches and leaflets exhibit an increase in calcium signaling (Ding et al., 2022). In a healthy hierarchy, this process are local sensors that filter synaptic activity, however, the increase in calcium activity due to aging introduce noise to the signal shifting away from localized calcium activity toward maladaptive and global responses.

## Future directions and conclusion

Astrocytes are active and dynamic elements of neural circuits. However, we still do not fully understand their computational power. Astrocytes are not uniform; instead, they are composed of multiple functional subdomains with processes differing in sensitivity, connectivity, and providing different influence on the soma activity. All this points to a hierarchical organization of information flow within an astrocyte. Astrocytes operate through specialized domains with some acting as high-efficiency input sites tuned for synaptic detection, while others serve as effector channels optimized for the dissemination of signals to the vasculature or specific synapses. This polarity ensures that information flux follows a weighted hierarchy, allowing astrocytes to filter, amplify, or redistribute signals according to circuit demands.

We hypothesize that this hierarchy is not static but dynamically shaped across the lifespan and during disease. During neurodevelopment, this polarity can be established by circuit maturation, in which specific processes would progressively acquire dominant roles. In the aged brain, the disruptions in astrocyte calcium activity would shift away from modular activity in microdomains signaling toward maladaptive centralized calcium responses. In pathological states such as epilepsy, this reconfiguration becomes even more pronounced; mitochondrial fission and oxidative stress can lower the threshold for global activation, short-circuiting the astrocyte’s internal dynamics and transforming local processing into whole cell activity. Future work should focus on mapping the real-time reconfiguration of astrocyte networks under physiological and pathological conditions.

Future work should focus on mapping the real-time reconfiguration of astrocytic networks under physiological and pathological conditions. Understanding how epilepsy, aging, or neurodegeneration short-circuit this hierarchy, transforming specialized local processors into disorganized global responders, will be essential for developing targeted interventions to restore the astrocyte’s role as active member of the tripartite synapse. Ultimately, defining how hierarchical input–output relationships are established, maintained, and disrupted will be crucial to reframe astrocytes not only as support cells but as dynamic processors whose organization shapes both healthy and diseased brain function and will be fundamental to our understanding of brain complexity.

## Data Availability

The data analyzed in this study is subject to the following licenses/restrictions: the data of the paper would be shared upon request. Requests to access these datasets should be directed to lopezhidalgo@unam.mx.
